# XBP1 is required in Th2 polarization induction in airway allergy

**DOI:** 10.7150/thno.75100

**Published:** 2022-07-11

**Authors:** Xianhai Zeng, Xiaojun Xiao, Suqin Hu, Weiyi He, Gaohui Wu, Xiaorui Geng, Jialiang Fan, Longpeng Ma, Jiangqi Liu, Zhiqiang Liu, Pingchang Yang

**Affiliations:** 1 Department of Otolaryngology. Longgang E.N.T Hospital & Shenzhen Key Laboratory of E.N.T, Institute of E.N.T Shenzhen, China.; 2 Guangdong Provincial Key Laboratory of Regional Immunity and Diseases, Shenzhen, China.; 3 Institute of Allergy & Immunology, Shenzhen University School of Medicine, State Key Laboratory of Respiratory Disease Allergy Division at Shenzhen University, Shenzhen, China.; 4 Department of Respirology and Allergy, Third Affiliated Hospital of Shenzhen University, Shenzhen, China.

**Keywords:** Airway allergy, air pollution, CD4^+^ T cells, Th2 polarization, XBP1.

## Abstract

**Rationale**: Th2 polarization plays a central role in the pathogenesis of allergic diseases such as airway allergy. The underlying mechanism is not fully understood yet. X-box-binding protein-1 (XBP1) can regulate immune cell activities upon exposing stressful events. The role of XBP1 in the development of Th2 polarization has not yet been explored.

**Methods**: Mice carrying *Xbp1*-deficient CD4^+^ T cells were employed to observe the role of XBP1 in the induction of airway allergy. A cell culture model was established to evaluate the role of XBP1 in facilitating the Th2 lineage commitment.

**Results:** We found that *Xbp1* ablation in CD4^+^ T cells prevented induction of Th2 polarization in the mouse airway tract. XBP1 was indispensable in the Th2 lineage commitment. XBP1 mediated the effects of 3-methyl-4-nitrophenol (MNP) on facilitating inducing antigen-specific Th2 response in the airways. Exposure to MNP induced expression of XBP1 in CD4^+^ T cells. RhoA facilitated the binding between XBP1 and GATA3 in CD4^+^ T cells. XBP1 induced GATA3 phosphorylation to promote the *Il4* gene transcription. Modulation of the RhoA/XBP1 axis mitigated experimental allergic response in the mouse airways.

**Conclusions**: A potential therapeutic target, XBP1, was identified in this study. XBP1 was required in the development of skewed Th2 response in the airways. Inhibiting XBP1 alleviated Th2 polarization-related immune inflammation in the airways. The data suggest that inhibiting XBP1 has the translation potential for the treatment of airway allergy.

## Introduction

Th2 aberrant polarization is central to the pathogenesis of many immune disorders, including allergic diseases, autoimmune diseases, and parasitic inflammation^1^. Th2 polarization indicates a phenomenon that Th2 cells aggregate in the local tissues, and produce a large unnecessary quantity of Th2 cytokines. A key pathogenic characteristic of allergic rhinitis (AR) is Th2 polarization^1^,^2^. For example, profound Th2 cell infiltration and Th2 cytokine production can be detected in the nasal mucosa and the nasal secretions of subjects with AR. This can be regarded as an indicator of the pathological conditions in the airways with allergic disorders^2^. Although this phenomenon has been observed for many years, the mechanism of aberrant development of Th2 polarization in AR subjects is still not fully understood.

There was general consensus that IL-4 plays an essential role in the Th2 response^2^. IL-4 is principally produced by CD4^+^ T cells. Exposure to IL-4 also causes naive CD4^+^ T cells to differentiate into Th2 cells^3^. GATA3 is a transcription factor for IL-4. Published data show that GATA3 levels are significantly increased in the *IL4* promoter locus of CD4^+^ T cells in patients with AR^4^. Ablation of GATA3 prevents the induction of IL-4 by T cells^5^. However, the underlying mechanism by which GATA3 increases in the *IL4* promoter is not fully understood yet. The factors facilitating GATA3 towards the nuclei still need to be clarified further.

Endoplasmic reticulum (ER) stress is known to be involved in the pathogenicity of allergic diseases^6^. X-box-protein-1 (XBP1) is an ER stress sensor. Its original form is XBP1u, and must be converted to XBP1s (XBP1, in short) after splicing^7^. Cumulative reports suggest that XBP1 is involved in the pathogenesis of malignant tumors^8^. It is found that XBP1s is also associated with immune inflammation^6^,^7^,^9^. Published data indicate that XBP1 promotes the expression of Th2 cytokine in a mTOR and MAPK-dependent manner^10^,^11^. Therefore, it is important to explore the role of XBP1 in developing Th2 polarization. However, it remains to be determined whether XBP1 participates in regulating GATA3 activities.

RhoA (a RAS analog) is involved in the pathogenesis of allergic diseases, such as asthma, allergic rhinitis and dermatitis^12^-^13^,^14^. RhoA belongs to the small GTPase family; its functional status can be regulated by both RAS activators and deactivators^15^. Ablation of RhoA shows effects on suppressing allergic diseases in animal model studies^16^. The role of RhoA in regulating GATA3 activities in the development of Th2 polarization needs to be clarified. In this study, we found that the ablation of XBP1 prevented the development of airway allergy. The presence of XBP1 and RhoA increased GATA3 activities in CD4^+^ T cells to trigger Th2 polarization.

## Materials and methods

### Human subjects

Patients with perennial allergic rhinitis (AR) and Healthy Control (HC) subjects were recruited for this study at the Longgang ENT Hospital. A written informed consent was obtained from each human subject. The study protocol was reviewed and approved by the Human Ethics Committee of the hospital. The diagnosis of AR was carried out by our physicians following the routine procedures that can be found elsewhere^17^. The inclusion criteria for patients were: perennial AR history more than 2 years; had AR clinical symptoms (paroxysmal nasal itch, sneezing, profound nasal discharge), serum antigen specific IgE positive [≥0.35 kU/L; assessed by ImmunoCap; (ThermoFisher Scientific, Uppsala, Sweden)], antigen skin prick test (SPT; referred to published procedures^18^) positive. Patients were advised not using any anti-AR medicines for one week prior to recruitment. The exclusion criteria include: nasal polyp, sinusitis, cancers, autoimmune diseases, severe organ diseases, taking immune suppressive agents for any reasons. The demographic data of human subjects are presented in Table 1. The sample size of human subject was calculated with a formula: N = Z^2^ × (P × (1 - P)) / E^2^, in which N is sample size; Z is statistical magnitude (when the confidence coefficient is 95%, Z = 1.96); E is error value; P is probability value.

### Human serum and peripheral blood mononuclear cell (PBMC) preparation

Blood samples were taken from each human subject through the ulnar vein puncture. A portion of the blood samples were kept at 4 °C overnight; the serum was then collected for further experiments. PBMCs were isolated from blood samples by gradient Percoll density centrifugation.

### Identification and isolation of antigen specific CD4^+^ T cells

PBMCs were incubated with an anti-GL7 (AF790) Ab for 2 h. The GL7^+^ cells were eliminated by flow cytometry (FCM). Remainder cells were cultured in the presence of specific antigen (1 µg/ml) overnight. Cells were stained with Abs of CD3 (AF546), CD4 (AF488), and GL7 (AF790). In FCM, CD3^+^ CD4^+^ cells were gated first, from which GL7^+^ cells were gated or sorted out, and used as antigen specific CD4^+^ T cells.

### Assessment of cell viability

Before each cell experiment, cell viability was evaluated using the blue Trypan exclusion test. Cell viability ranged from 98% to 100% throughout the relevant experiments.

### Statistics

The difference between two groups was determined by Student *t* test (for normal distribution data) or Mann Whitney test (for non-normal distribution data). Multiple comparisons were performed in the data from more than 2 groups followed by Dunnett's test or Bonferroni test. The correlation between data obtained from two groups was tested by Spearman correlation coefficient test (for non-normal distribution data) or Pearson correlation coefficient test (for normal distribution data). P < 0.05 was considered statistically significant.

### Description of supplemental materials

The supplemental materials include a portion of materials and methods, and supplemental figures: Reagents, development of Th2 polarization in the mouse airways, cell culture, flow cytometry, enzyme-linked immunosorbent assay, assessment of serum Derf1 specific IgE (sIgE) by ELISA, mice, real-time quantitative RT-PCR, preparation of protein extracts, Western blotting, immunoprecipitation, chromatin IP, isolation immune cells, Th2 polarization, RNA interference (RNAi), mass spectrometry (MS), plasmids of *XBP1*, *GATA3* and *RHOA,* luciferase assay, and confocal microscopy.

## Results

### XBP1 levels in antigen specific Th2 cells are positively correlated with Th2 response in patients with allergic rhinitis (AR)

**Figure 1:**
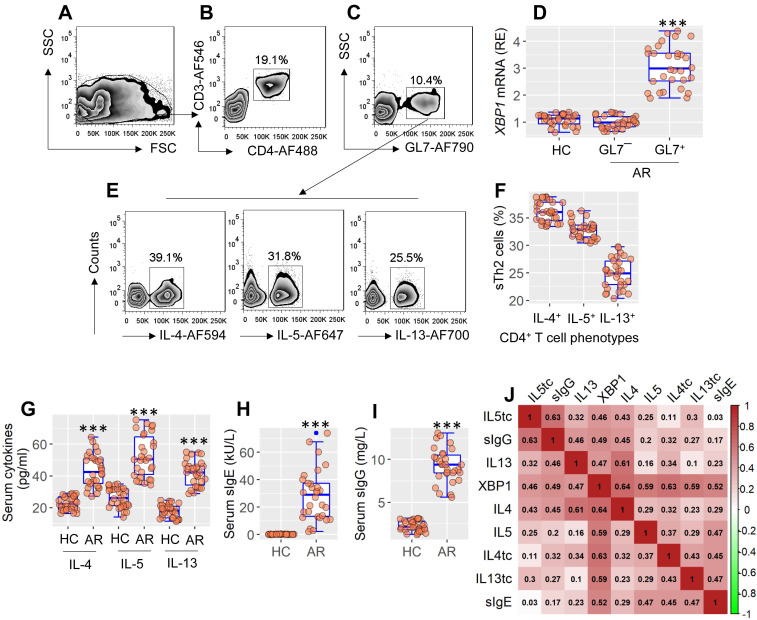
** XBP1 in sTh2 cells is associated with Th2 polarization**. PBMCs were isolated from blood samples of AR patients (n = 30) and health control (HC) subjects (n = 30). Pre-existing GL7^+^ cells in PBMCs were pre-cleared by MACS. Remainder cells were cultured with specific antigens (mite extracts) overnight, and analyzed by flow cytometry (FCM). A, FCM FSC/SSC plots. B, the CD3^+^ CD4^+^ T cells were gated. C, GL7^+^ cells (activated cells) and GL7¯ cells (non-activated cells) were purified by FCM, and analyzed by RT-qPCR. D, boxplots show the *XBP1* mRNA levels in GL7^+^ cells and GL7¯ cells. E, gated FCM plots show phenotypes of antigen specific Th2 cells (sTh2 cells). F, boxplots show sTh2 cell counts of 30 samples per group. G-I, boxplots show serum levels of Th2 cytokines, sIgE and sIgG. J, heatmap show correlation coefficients between *XBP1* mRNA levels in sTh2 cells and Th2 response. tc: T cells. ***p < 0.001, compared with the HC group (ANOVA + Dunnett's test for D; *t* test for G, H, I). In panel J, **p < 0.01, ***p < 0.001 (Spearman correlation coefficient assay). Numbers in heatmap are coefficients. AF: Alexa Fluor. PBMC: Peripheral mononuclear cells. FCM: Flow cytometry. MACS: Magnetic cell sorting. sIgE: Specific IgE. AR: Allergic rhinitis.

Published data indicate that XBP1 may be linked to allergic disorders^11^, but the detailed mechanism remains to be explored. To this end, blood samples were taken from patients with allergic rhinitis, from which antigen-specific CD4^+^ T cells were isolated, and analyzed by RT-qPCR and flow cytometry (FCM). Expression of XBP1 was significantly higher (p < 0.001) in antigen-specific CD4^+^ T cells than in nonspecific CD4^+^ T cells or CD4^+^ T cells in HC subjects (Figure 1A-F). Patients with AR exhibited the Th2 polarization characteristics by showing a high frequency of antigen-specific Th2 cells (sTh2 cells) (Figure 1E-F), higher (p < 0.001) serum Th2 cytokines (Figure 1G), serum specific IgE, and sIgG. (Figure 1H-I). Th2 polarization parameters were positively correlated with the *XBP1* mRNA levels in sTh2 cells (Figure 1 I). The data suggest that XBP1 in Th2 cells may play a key role in the development of Th2 polarization.

### XBP1 ablation in CD4^+^ T cells prevents Th2 polarization induction in the airways

**Figure 2:**
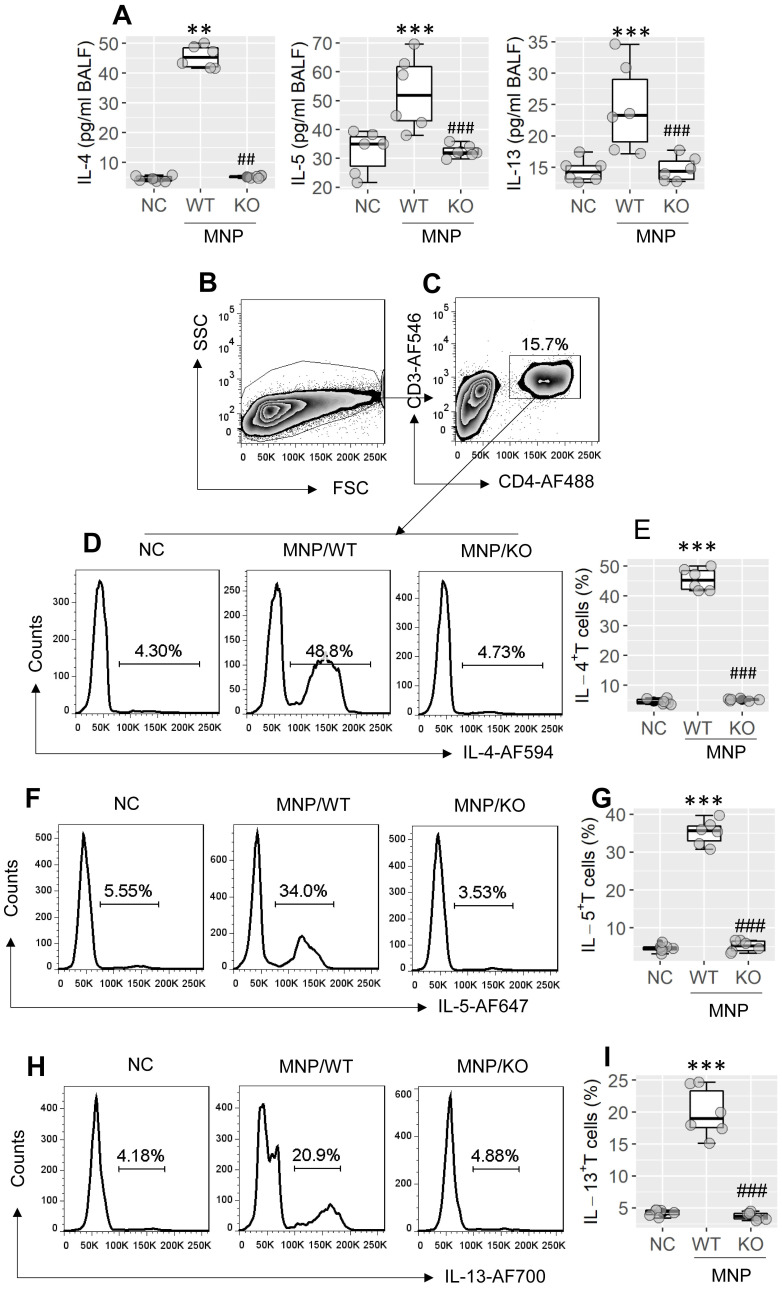
** Assessment of the role of XBP1 in Th2 polarization development in the airway tissues**. Wild type (WT) mice and *Xbp1*^∆CD4^ mice (KO mice, mice carrying *Xbp1*-deficient CD4^+^ T cells) (6 mice per group) were treated with MNP through nasal instillation daily for 2 weeks. BALF was collected from each mouse after the last treatment with MNP. A, boxplots show cytokine levels in BALF of mice. B-I, cells were isolated from BALF, and analyzed by FCM. B, the FSC/SSC plots. C, CD3^+^ CD4^+^ T cells were gated. D-I, gated FCM plots show Th2 cell (IL-4^+^, IL-5^+^, IL-13^+^) counts; boxplots show Th2 cell frequency in cells of BALF. **, p < 0.01, ***, p < 0.001, compared with the NC (naïve control) mice (ANOVA + Dunnett's test). ##, p < 0.01, ###, p < 0.001, compared with the WT/MNP group (Student *t* test). Each dot in boxplots presents data obtained from one mouse. MNP: 3-methyl-4-nitrophinol. FCM: Flow cytometry.

Referring to published strategy^19^,^20^, a mouse strain with the *Xbp1* gene conditionally knockout (KO) in CD4^+^ T cells (the *Xbp1*^f/f^ *CD4*^Cre^ mice; *Xbp1*^∆CD4^ mice, in short) was generated. The number of bone marrow cells was unchanged between WT mice and *Xbp1*^∆CD4^ mice (Figure S1A in supplemental materials). In contrast to wild type (WT) mice (C57/B6 mice), which showed the XBP1 expression in CD4^+^ T cells, the *Xbp1* mRNA was not detected in CD4^+^ T cells isolated from *Xbp1*^∆CD4^ mice (Figure S1B). The ablation of *Xbp1* did not impact on the expression of inositol-requiring enzyme (IRE1α) (Figure S1C). Total number of CD4^+^ T cell, B cell, macrophage, dendritic cell (DC), Th1 cells and Th17 cells in the spleen, intestine and airway tissues were not apparently altered in *Xbp1*^∆CD4^ mice (Figure S1D-I; p > 0.05). A mouse model of Th2 polarization in airway tissues was developed using MNP through nasal instillation following the published protocol^21^ (the MNP protocol, in short; Figure S2A). We observed that the Th2 polarization status was induced in the airway tissues of sensitized WT mice, manifesting the increase in Th2 cytokines [IL-4 (p < 0.01), IL-5 (p < 0.001), IL-13 (p < 0.001)] in bronchoalveolar lavage fluids (BALF) (Figure 2A), and increase in Th2 cells in BALF (Figure 2B-I, p < 0.001), which did not occur in *Xbp1*^∆CD4^ mice (Figure 2A-I). The results suggest that XBP1 in CD4^+^ T cells plays a central role in the development of Th2 polarization in the airways.

### XBP1 is indispensable in the Th2 lineage commitment

**Figure 3:**
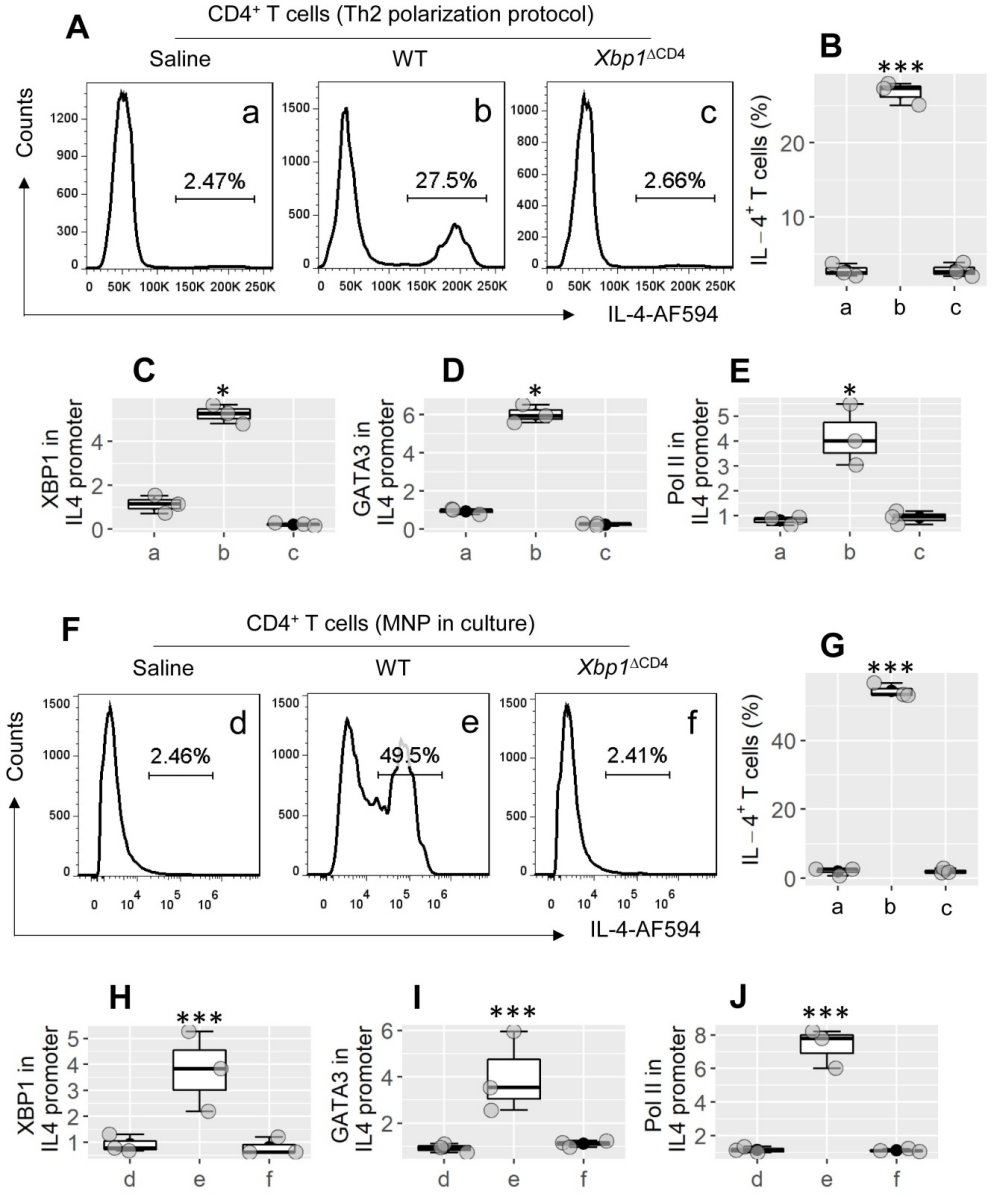
** XBP1 is required in Th2 cell development**. CD4^+^ CD62L^+^ T cells were isolated from the spleen of WT mice and *Xbp1*^∆CD4^ mice, and treated with the Th2 polarization protocol (A-E) or adding MNP (1 μM) to culture (F-J). The gated FCM histograms show IL-4^+^ cell counts (A). The boxplots in B and G show IL-4^+^ cell frequency. C-E and H-J, CD4^+^ T cells were harvested at the end of culture; cellular extracts were prepared and analyzed by ChIP. Boxplots of C-E show levels of XBP1 (C), GATA3 (D) and Polymerase II (Pol II, E) against input (fold change) in ChIP products. Each dot in boxplots presents data obtained from one sample. *p < 0.05, *** p < 0.001 (ANOVA + Dunnett's test), compared with group a. The data in A are from one experiment that represent 3 independent experiments. Group labels of C-E and H-J are the same as those in FCM plots. WT: Wild type. *Xbp1*^∆CD4^ mice: Mice carry *Xbp1*-deficient CD4^+^ T cells. MNP: 3-methyl-4-nitrophenol. FCM: Flow cytometry. ChIP: Chromatin immunoprecipitation.

To evaluate the role of XBP1 in the bioactivity of Th2 cells, CD4^+^ CD62L^+^ T cells were isolated from the spleen of WT mice and *Xbp1*^∆CD4^ mice. The cells were treated with the Th2 polarization protocol in culture. As shown by Figure 3A-E, CD4^+^ T cells were converted to Th2 cells from WT CD4^+^ T cells by treating with the Th2 cell polarization protocol in culture, but not in *Xbp1*^ΔCD4^ CD4^+^ T cells (Figure 3A-B; p < 0.001). The results suggest that XBP1 is required in the development of Th2 cells, probably involving in the transcription of the *Il4* gene. By chromatin immunoprecipitation (ChIP) assay, we observed both XBP1 and GATA3, and high levels of polymerase II (Pol II; an indicator of gene transcription) in the *Il4* promoter locus of WT CD4^+^ T cells after treating cells with the Th2 polarization protocol; this phenomenon did not occur in *Xbp1*^ΔCD4^ T cells (Figure 3C-E). Since exposure to MNP (3-methyl-4-nitrophenol; an environmental pollutant) induces Th2 polarization in the airways (Figure 2), we then tested the role of MNP in the induction of *Il4* transcription in CD4^+^ T cells. In fact, exposure to MNP in culture led to a significant increase (p < 0.001) in the transcription of the *Il4* gene and the development of IL-4^+^ T cells (Figure 3F-J). The results demonstrate that XBP1 is indispensable in the *Il4* gene transcription in CD4^+^ T cells.

### XBP1 mediates the effects of MNP on facilitating antigen-specific Th2 response in the airways

**Figure 4:**
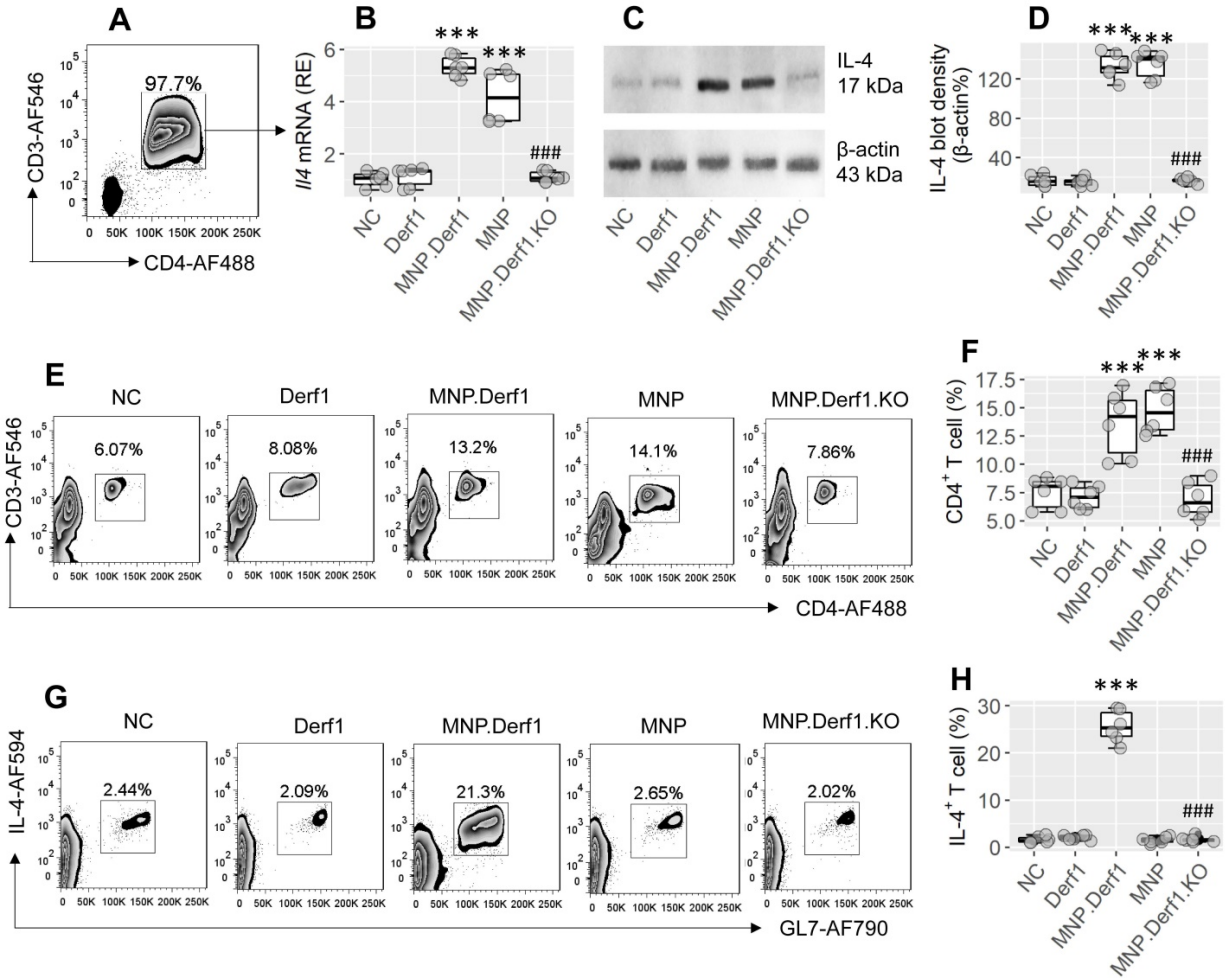
** Assessment of the role of XBP1 in the MNP-facilitated antigen-specific Th2 response development in the airways**. Mice were treated with Derf1 or/and MNP as depicted in Fig. S2B. A-B, gated FCM plots show purified CD3^+^ CD4^+^ T cells isolated from the airway tissues. Boxplots show the IL-4 mRNA levels. C-D, immunoblots show protein levels in isolated CD4^+^ T cells; boxplots show integrated density of the immunoblots. E-F, gated FCM plots show CD3^+^ CD4^+^ T cell counts and boxplots show the CD4^+^ T cell frequency in the airway tissues. G-H, purified CD4^+^ T cells were cultured in the presence of Derf1 (the specific antigen; 30 µg/mL) and DCs overnight, and analyzed by FCM. Gated FCM plots show the activated Th2 cell counts. Boxplots show the activated Th2 cell frequency. ***, p < 0.001 (ANOVA + Dunnett's test), compared with the NC (naïve control) group. ###, p < 0.001 (*t* test), compared with the MNP.Derf1 group. MNP.Derf1.KO: *Xbp1*^∆CD4^ mice were treated with the MNP.Derf1 protocol. (P.S., exposure to ovalbumin, an irrelevant antigen, in the culture did not induce the Th2 response. Data not shown). MNP: 3-methyl-4-nitrophenol. FCM: Flow cytometry. Derf1: D. *farinaeallergen* allergen 1.

We then treated mice with MNP or/and Derf1 (a major allergen protein of house dust mites), as presented in the MNP.Derf1 protocol (Figure S2B). We found that *Il4* mRNA and IL4 protein were markedly increased (p < 0.001) in CD4^+^ T cells isolated from the airway tissues of the MNP group and the MNP.Derf1 group, but not the Derf1 alone group (Figure 4A-D). Although exposure to either MNP or MNP.Derf1 increased CD4^+^ T cells in the mouse airway tissues, the antigen-specific Th2 cells were induced in the airways of the MNP.Derf1 group, but not in the MNP alone group (Figure 4E-H). Ablation of XBP1 in CD4^+^ T cells abolished the induction of antigen-specific Th2 cells in the airways (Figure 4G-H). The results indicate that concomitant exposure to MNP and Derf1 induces antigen-specific Th2 response in the airway tract, where XBP1 is required.

### RhoA mediates the effects of MNP on the induction of XBP1 expression in CD4 T cells

**Figure 5:**
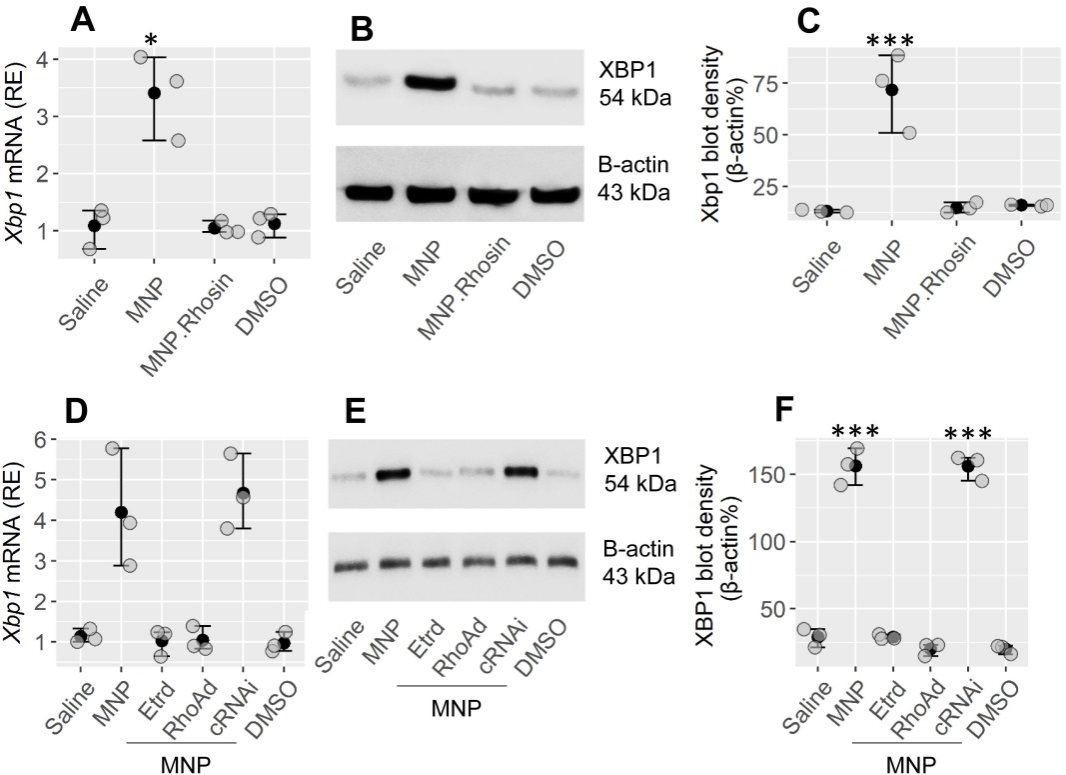
** Exposure to MNP induces XBP1 expression in CD4^+^ T cells**. A-C, mice were treated with the reagents listed on the x axis of panel A through nasal instillation [MNP: 2.5 mg/mL; Rhosin (an inhibitor of RhoA): 1 μM; DMSO (solvent): 0.5 μl/mL] daily for 2 weeks. CD4^+^ T cells were isolated from the airway tissues and analyzed. A, the mRNA levels of XBP1 in CD4^+^ T cells. B, the protein levels of XBP1 in CD4^+^ T cells. C, integrated density of immunoblots in panel B. D-F, naïve spleen CD4^+^ T cells were exposed to the reagents (MNP: 1 μM; DMSO: 0.5 μl/mL; Etr: Estrogen receptor. Etrd: Etr-deficient CD4^+^ T cells; RhoAd: RhoA-deficient CD4^+^ T cells; cRNAi: CD4^+^ T cells were treated with scramble shRNA) listed on the x axis of panel D for 24 h. D, the mRNA levels of XBP1 in CD4^+^ T cells. E, the protein levels of XBP1 in CD4^+^ T cells. F, integrated density of immunoblots in panel B. *p < 0.05, **p < 0.01, ***p < 0.001, compared with the saline group (ANOVA + Dunnett's test. The data represent 3 independent experiments. MNP: 3-methyl-4-nitrophenol.

Our previous studies have shown that exposure to MNP, a major environmental pollutant, can facilitate the development of allergic reactions^21^. This prompted us to look for a probability that XBP1 could be involved in the development of MNP-facilitated Th2 polarization in the airways. The CD4^+^ T cells isolated from the mouse and spleen airways demonstrated the expression of estrogen receptor-α (Etr) (Figure S3A). Mice were treated with MNP by nasal instillation daily for 2 weeks (Figure S2A). CD4^+^ T cells were isolated from the airway tissues. Higher levels of XBP1 were found in isolated CD4^+^ T cells in MNP-treated mice than in saline-treated control mice (Figure 5A-C). The results indicate that MNP exposure induces XBP1 expression in CD4^+^ T cells. RhoA mediates the effects of MNP on modulation of target cell activity^22^. Mice were treated with MNP with or without Rhosin (Rechembioscience, Shanghai, China; a RhoA GTP inhibitor^23^). In fact, Rhosine administration inhibited the XBP1-induced expression in CD4^+^ T cells (Figure 5A-C). To verify the findings, naive CD4^+^ T cells were isolated from the spleen, and exposed to MNP in culture for 24 h. We found that exposure to MNP markedly induced the XBP1 expression in CD4^+^ T cells, which was abolished by knocking down either Etr or RhoA in CD4^+^ T cells (Figure S3B-E, Figure 5D-F). The results demonstrate that exposure to MNP may be one of the factors that cause XBP1 expression in CD4^+^ T cells.

### RhoA facilitates the binding between XBP1 and GATA3 in Th2 cells

**Figure 6:**
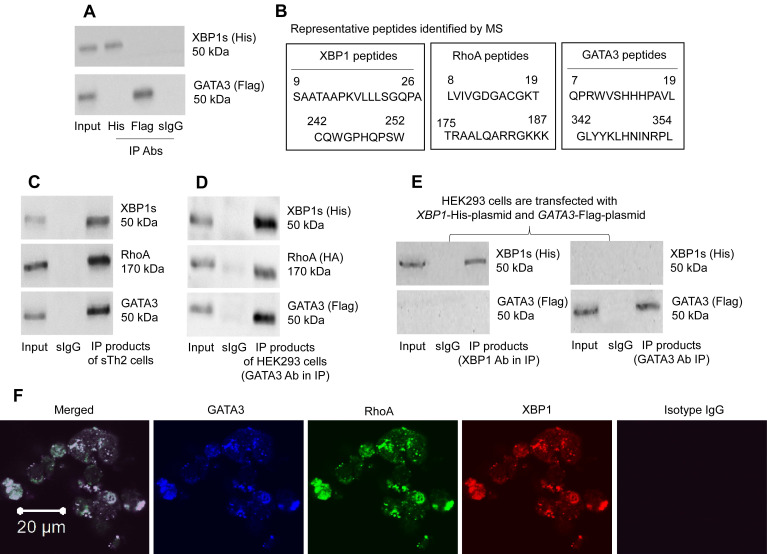
** RhoA, XBP1, GATA3 form a triple complex**. A, His-*XBP1* plasmids and Flag-*GATA3* plasmids were transfected into HEK293 cells, from which cell extracts were prepared, and analyzed by co-IP. Immunoblots show the complex of XBP1/GATA3 was not detected in HEK293 cells. B, antigen-specific Th2 cells were isolated from the airway tissues of mice immunized with the Derp1.MNP protocol, from which cell extracts were prepared and precipitated with an anti-GATA3 Ab as the IP Ab. IP products were analyzed by mass spectrometry (MS). The amino acid sequences show representative peptides of GATA3, XBP1 and RhoA, respectively. C, immunoblots show a triple complex of GATA3, XBP1 and RhoA in IP products with extracts of sTh2 cells. D, plasmids of *XBP1*, *GATA3* and *RHOA* were transfected to HEK293 cells, from which cell extracts were prepared, and analyzed by IP with an anti-GATA3 as the IP Ab. Immunoblots show a triple complex of GATA3, XBP1 and RhoA. E, plasmids of *XBP1* and *GATA3* were transfected to HEK293 cells, from which cell extracts were prepared, and analyzed by IP with an anti-GATA3 or anti-XBP1 as the IP Ab. Immunoblots show GATA3 and XBP1 do not form a complex in HEK293 cells. F, confocal images (×630) show a complex of GATA3, RhoA and XBP1 in HEK293 cells. The data represent 3 independent experiments. IP: Immunoprecipitation. Input: Protein sample before IP.

The data of Figure 2-4 suggest that XBP1 may interact with GATA3 to facilitate the *IL4* gene transcription. To check if XBP1 has physical contact with GATA3 in CD4^+^ T cells, *XBP1*-His-CMV plasmids and *GATA3*-Flag-CMV plasmids (Figure S5) were transfected into HEK293 cells. However, the predicted XBP1/GATA3 complex was not detected in HEK293 cells as shown by IP products (Figure 6A). Considering that there could be substances facilitating the formation of the XBP1/GATA3 complex, we isolated the CD4^+^ T cells from the airway tissues of mice immunized with the MNP.Derf1 protocol (Figure S2), from which the Derf1-specific Th2 cells (sTh2 cells) were purified using the specific antigen exposure protocol (Figure S4). Protein extracts were prepared with purified sTh2 cells, and analyzed by Immunoprecipitation (IP) with an anti-GATA3 Ab as a precipitation Ab. The IP products were analyzed using mass spectrometry (MS). The MS results showed that, apart from the presence of GATA3, XBP1 and RhoA were detected in the IP products (Figure S6, Figure 6B). The results suggest that RhoA could be the factor facilitating the formation of XBP1 and GATA3 complexes in CD4^+^ T cells. The inference was verified by that a triple GATA3/XBP1/RhoA complex was detected in the sTh2 cells (Figure 6C), but not in naïve CD4^+^ T cells (not shown). Furthermore, *XBP1*-His-CMV plasmids, *GATA3*-Flag-CMV plasmids and *RHOA*-HA-CMV plasmids were transfected into HEK293 cells. Protein extracts were prepared with the HEK293 cells, and analyzed by IP with an anti-Flag (GATA3) Ab as a precipitation Ab. A triple complex of His-XBP1/HA-RHOA/Flag-GATA3 was detected (Figure 6D). However, transfected *XBP1*-His-CMV plasmids and *GATA3*-Flag-CMV plasmids into HEK293 cells did not form a complex (Figure 6E). The complex in HEK293 cell was also illustrated by confocal microscopy data (Figure 6F). The results demonstrate that RhoA facilitates the formation of XBP1/GATA3 complex in cells.

### XBP1 facilitates GATA3 phosphorylation for activating promoter *Il4*

**Figure 7:**
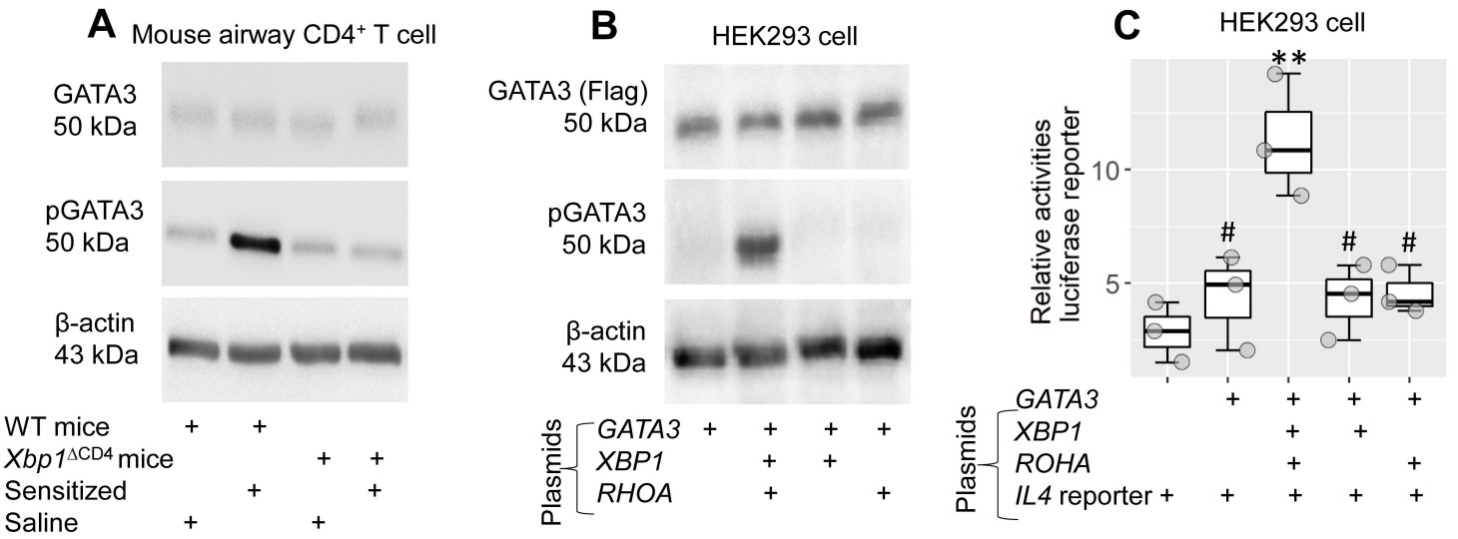
** The role of XBP1 in facilitating *IL4* promoter activation**. A, WT mice and *Xbp1*^∆CD4^ mice were sensitized with the MNP.Derf1 protocol (Fig. S2). CD4^+^ T cells were purified from the airway tissues (sTh2 cells were further purified from CD4^+^ T cells isolated from sensitized mice), cytosolic and nuclear proteins were prepared with the cells, and analyzed by Western blotting. Immunoblots show the levels of GATA3 and phosphorylate GATA3 (pGATA3, in the nuclei) in CD4^+^ T cells of mice after the treatment denoted below immunoblots. B, *GATA3* plasmids with or without *XBP1* plasmids and *RHOA* plasmids were transfected into HEK293 cells. Cellular extracts were prepared, and analyzed by Western blotting. Immunoblots show the levels of GATA3 and pGATA3. C, an *IL4* promoter luciferase reporter together with plasmids of GATA3, XBP1 and RHOA (as denoted on the x axis). Boxplots show luciferase activities in different combination of the plasmids. Statistical method: ANOVA + Dunnett's test. **p < 0.01, compared with the group of *IL4* promoter alone group. #, p < 0.05, compared with the data obtained from HEK293 cells transfected with all the 4 plasmids. The data represent 3 independent experiments. MNP: 3-methyl-4-nitrophenol. Derf1: D. *farinaeallergen* allergen 1. WT: Wild type. *Xbp1*^∆CD4^ mice: Mice carry *Xbp1*-deficient CD4^+^ T cells.

Current data indicate that XBP1 is needed to facilitate the effects of GATA3 on transcription of the *Il4* gene in CD4^+^ T cells. To better understand the mechanism behind it, WT mice and *Xbp1*^∆CD4^ mice were treated with the MNP.Derf1 protocol to develop airway allergy. The mice were designated “sensitized mice” (data of the airway allergy response not shown). CD4^+^ T cells were isolated from the airway tissues, from which sTh2 cells were purified in the sensitized group, and analyzed by Western blotting. Phosphorylated GATA3 levels were significantly higher in sTh2 cell nuclear extracts of sensitized WT mice than that from CD4^+^ T cells of *Xbp1*^∆CD4^ mice (Figure 7A). The results suggest that XBP1 facilitates the phosphorylation of GATA3 during sensitization. To test this, plasmid *GATA3*-Flag-CMV or *GATA3*-Flag-CMV, *RhoA*-HA-CMV and *XBP1*-His-CMV were transfected in HEK293 cells. Cellular extracts were prepared and analyzed using Western blotting. Transfection of GATA3 plasmids alone induced GATA3 expression, but yielded no pGATA3, in HEK293 cells. Transfection of *GATA3*-Flag-CMV plasmids together with *RhoA*-HA-CMV plasmids and *XBP1*-His-CMV plasmids resulted in both GATA3 and pGATA3 in HEK293 cells. The absence of XBP1 or RhoA plasmids failed to induce GATA3 phosphorylation (Figure 7B). Since the role of RhoA is to facilitate the link between XBP1 and GATA3, the results underline the role of XBP1 in the promotion of GATA3 phosphorylation. Given that GATA3 is the transcription factor of *IL4*, the results imply that physical contact between GATA3 and XBP1 is required to activate the *IL4* promoter. To test this, an *IL4* promoter plasmid reporter (carrying luciferase) was transfected with *GATA3*, *XBP1* and *RhoA* plasmids in HEK293 cells. As shown in the luciferase assay, transfection with GATA3 alone did not increase luciferase activities in HEK293 cells. Transfection with *GATA3*, *XBP1* and *RhoA* plasmid significantly increased (p < 0.01) luciferase activity. Results were not obtained without *GATA3*, *XBP1* or *RhoA* plasmid transfection (Figure 7C).

### Modulation of the RhoA/XBP1 pathway reduces the experimental allergic response in the airways

**Figure 8:**
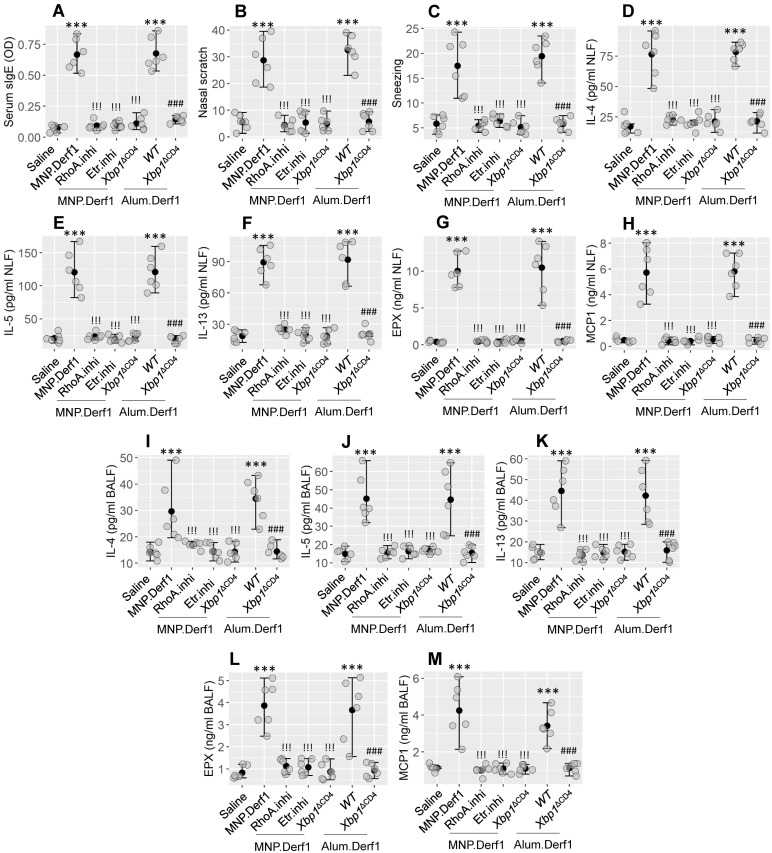
** Modulating the RhoA/XBP1 axis inhibits airway allergy**. Airway allergy mouse models were development with the Bap.Derf1 protocol or the Alum.Derf1 protocol. The airway allergy response was recorded, including serum specific IgE (sIgE) levels (A), nasal allergy clinical symptoms (nasal itch and sneezing, B-C), Th2 cytokines and allergic mediators in nasal lavage fluid (NLF; D-H) and bronchoalveolar lavage fluid (BALF; I-M). Etr.inhi (Etr: Estrogen receptor. “inhi” stands for “inhibitor”): Etr inhibitor. Mice were treated with nasal instillation containing Bay-2416964 (1 μM). RhoA.inhi: Mice were treated with nasal instillation (20 μl/nostril, containing RhoA inhibitor, Bay-293 40 nM) daily during the sensitization period. *Xbp1*^ΔCD4^: Mice carry *Xbp1*-deficient CD4^+^ T cells. WT: C57B/6 mice (littermates of *Xbp1*^∆CD4^ mice). Each group consists of 6 mice. Each dot presents data obtained from one mouse. The data are presented as median (IQR). *** (p < 0.001; ANOVA + Dunnett's test), compared with the saline group. !!! (p < 0.001; ANOVA + Dunnett's test), compared with the MNP.Der1 group. ### (p < 0.001; *t* test), compared with the WT (alum.Derf1) group. The experiments were repeated 3 times. MNP: 3-methyl-4-nitrophenol. Derf1: D. *farinaeallergen* allergen 1.

Then we developed an airway-allergic mouse model using the MNP.Derf1 protocol (Figure S2C). The immunized mice showed an increase in Derf1-specific IgE in the serum and in the airway allergy response, as shown in Figure 8, including airway allergic clinical symptoms (nasal itch and sneezing), increase in Th2 cytokines (IL-4, IL-5, and IL-13) and allergic mediators (EPX and MCP1) in nasal lavage fluids (NLF) and bronchoalveolar lavage fluids (BALF). The inhibition of either Etr or RhoA, or the depletion of *XBP1* in CD4^+^ T cells, prevented the induction of airway allergy using the MNP.Derf1 protocol. To expand the findings, WT mice and *Xbp1*^ΔCD4^ mice were treated with the Alum.Derf1 protocol. The airway allergic response was detected in WT mice, but not in *Xbp1*^ΔCD4^ mice. Additionally, airway mononuclear cells (AMC) were isolated from airway tissues, and analyzed by FCM. The results showed that Th2 cells were significantly increased in the airway tissues in mice sensitized by the MNP.Derf1 protocol. Ablation of *Xbp1*, inhibition of Etr or RhoA markedly inhibited the increase in Th2 cells in the airway tissues (Figure S7). The results indicate that XBP1 may be a therapeutic target for the treatment of airway allergies.

## Discussion

This study identified a new therapeutic target, XBP1 in CD4^+^ T cells, for airway allergy. The results show that the depletion of XBP1 abolished the induction of Th2 cells from naïve CD4^+^ T cells. This is shown by the results that XBP1 depletion eliminated induction of Th2 cells from naive CD4^+^ T cells. Exposure of CD4^+^ T cells to MNP, a major component of air pollution, resulted in XBP1 and RhoA being expressed in CD4^+^ T cells. RhoA facilitated the binding between XBP1 and GATA3, which moved to the *IL4* promoter in CD4^+^ T cells to induce *IL4* gene transcription and Th2 lineage engagement. It drives CD4^+^ T cells to produce IL-4, and become Th2 cells. The data suggest that XBP1 and RhoA are potential new targets for the treatment of airway allergy.

The data show the essential role of XBP1 in the induction of Th2 cells. XBP1 is an ER stress sensor. Earlier reports indicate that XBP1 promotes tumor growth by regulating the properties of dendritic cells 7. Regulation of XBP1 reduces IFN-γ production and decreases T cell infiltration into tumor tissues 20. Our data demonstrate that XBP1 is needed for Th2 lineage engagement and Th2 cell development. Th2 cells are a canonical fraction of T cells that play a central role in immune homeostasis in the body. A significant role of Th2 cells is the triggering of adaptive immunity. Others found that XBP1 was involved in the maintenance of immune cell functions. For example, Kaser et al reported that XBP1 was required to maintain bowel epithelial cell functions. XBP1 abnormality in the intestinal epithelial cells can cause organ-specific inflammation 9. Our data show another functional aspect of XBP1 in CD4^+^ T cells, which causes more Th2 cell development. Too many Th2 cells in local tissues can produce more Th2 cytokines than needed, which can contribute to the development of allergic disorders in the presence of specific allergens.

The data show that MNP exposure induces the expression of XBP1 in CD4^+^ T cells. MNP is one of the environmental pollutants present in the air or in most areas of the world. MNP can bind the estrogen receptor to activate target cells^24^. T cells express estrogen receptors. Activation of estrogen receptors on T-cells contributes to inflammation of the immune system^25^. Current results suggest that CD4^+^ T cells of individuals living in districts contaminated with MNP may be easily induced the expression XBP1. This is an interesting topic that needs to be explored further. Exposure to MNP can induce ER stress^26^, that may result in accumulation of unfolded protein response and cause cell apoptosis^26^. Consequently, it results in more XBP1 activities^7^. Increasing the expression of XBP1 is a response to counteract ER stress to keep homeostasis in the cell. However, cumulative reports indicate that XBP1 plays an important role in the pathogenesis of malignant tumors^27^ or immune inflammation^9^. Many causative factors of asthma can induce ER stress in the airway tissues^28^. ER stress induces more XBP1 expression 7 in CD4^+^ T cells^29^, that intends to cause Th2 polarization in the local tissues such as asthma^30^. Our study extended the study on XBP1 by showing that XBP1 is involved in the development of Th2 cells; it probably plays a crucial role in the development of Th2 polarization. With the presence of specific antigen, overexpression of XBP1 may lead to the development of allergy disorders. The implication is supported by current data. Exposure to MNP polarizes the Th2 response, whereas exposure to MNP and specific antigens induces an allergic reaction in the tissues of the airways.

The data show that RhoA acts as a mediator in MNP-induced XBP1 in CD4^+^ T cells. RhoA belongs to the small GTPase family, is one of the analogs of RAS. One of the biological features of the RAS family is RAS.GTP (activation status) and RAS.GDP (inactivation status) can automatically transform one another in a strictly regulated cycle. This cycle can be disturbed by unknown factors^31^. For example, in cancer cells, the status of RAS.GTP is sustained, that causes cancer cell growth out of control^32^. A similar phenomenon was observed in the current study. Levels of RhoA GTP are significantly higher in antigen-specific Th2 cells isolated in AR patients than in healthy individuals. This indicates that antigen-specific Th2 cells in patients with AR have a higher RhoA activation status. The results point to a link between this phenomenon and the development of Th2 cells and the strong expression of Th2 cytokines. By administering a RhoA inhibitor, MNP-induced Th2 cell polarization in the airways was suppressed. The results suggest that RhoA in CD4^+^ T cells can be a potential target for the treatment of allergic disorders involved in Th2 polarization.

The data also show mechanistic evidence whereby XBP1 facilitates the development of Th2 polarization in the airways. XBP1 is a transcription factor; not surprisingly, XBP1 is present in the promoters. To our knowledge, this is the first report that describes XBP1 with GATA3 in the *IL4* promoter in CD4^+^ T cells. It is the consensus that GATA3 is the transcription factor of *IL4*. However, co-transfection of *GATA3* and *IL4* plasmids in HEK293 cells did not activate the *IL4* promoter in the current experimental context. This phenomenon suggests that in addition to GATA3, the *IL4* gene transcription requires additional supports. Our data show that XBP1 is one of those supports in the transcription of the *IL4* gene. As the overproduction of IL-4 plays a central role in the pathogenesis of allergic diseases, modulation of IL-4 expression can mitigate disorders involving Th2 polarization. Current evidence suggests that XBP1 may be a new therapeutic target for allergic illnesses.

The data also suggest that RhoA participates in the transcription of the *IL4* gene. RhoA is required in the formation of the GATA3 and XBP1 complex in the *IL4* promoter of CD4^+^ T cells after activation. Previous studies also noted the involvement of RhoA in the pathogenesis of allergic diseases. Yang et al discovered that RhoA played an essential role in expressing IL-17 in the asthma process by orchestrating glycolysis in Th2 cells. Ablation of RhoA could alleviate experimental asthma^16^. Lovastatin is a cholesterol-lowering medication, which can be used to attenuate airway hyperresponsiveness in experimental asthma with a mechanism that inhibits RhoA signals in the airway^33^. Our previous work showed that RhoA had the effects on inducing airway allergy in mice through promoting the oxidative stress in epithelial cells^21^. Activation of the estrogen receptor can enable RhoA signaling^34^, which can trigger the allergic inflammatory pathway^16^. The present data show another functional characteristic of RhoA, which indicates that RhoA participates in the transcription of the *IL4* gene. This finding is significant, which show that the induction of Th2 cells can be prevented in the absence of RhoA in a cell culture system. In other words, the current study identified that RhoA was also a potential therapeutic target for airway allergy in addition to XBP1.

It is known that XBP1 regulates MHC class II genes by binding to a promoter element referred to as an X box^35^. It has been suggested to enhance viral protein expression by acting as a DNA binding partner of a viral transactor^12^. Hence, targeting XBP1 may also lead to off-target effects and alter the MHC-II expression important for immune responses against various infections.

It is known that only spliced correctly, XBP1 can activate unfolded protein response effectively to avoid apoptosis of host cells. Activating transcription factor 6 (ATF6), IRE1α and protein kinase RNA-like ER kinase (PERK) are involved in XBP1 mRNA splicing^36^. PERK mediates the IL-25-induced airway epithelial cell apoptosis to contribute to allergy development^37^. ATF6 plays a role in airway remodeling^38^. IRE1α favors Th2 response^39^. These data further confirm that XBP1 is involved in the pathogenesis of allergic response. It suggests that inhibition of PERK or ATF6 or IRE1α can also benefit to suppress allergic disorders, it is an interesting topic and can be studied further.

In conclusion, this study identified a potential therapeutic target for treating airway allergy. The data show that XBP1 plays a crucial role in inducing Th2 polarization in the airways. RhoA is necessary in XBP1-induced *Il4* gene transcription. Blocking XBP1 or RhoA can inhibit experimental airway allergy, suggesting that blocking RhoA or XBP1 has translation potential for treating airway allergy.

## Supplementary Material

Supplementary methods and figures.

## Figures and Tables

**Table 1:** Demographic data of human subjects

Item	AR	Healthy
Male/female	15/15	15/15
Age	32.3 ± 5.5	31.3 ± 5.8
Weight	58.6 ± 5.6	57.2 ± 8.2
Height	159.6 ± 6.7	157.5 ± 8.5
BMI	22.7 ± 2.4	23.1 ± 2.2
AR AR/asthma	30 (100%) 3 (10%)	00
AR/eczema	3 (10%)	0
Total IgE (kU/L)	0.22 (0.05, 0.33)*	0.31(0.08, 0.34)
Specific IgE (kU/L)	18.6 (0.56, 68.8)	nd
Current smoker	3 (10%)	3 (10%)
Blood neutrophil (10^9^/L)	4.55 (4.02, 5.86)	4.48 (4.24, 6.06)
Blood eosinophil (10^9^/L)	0.35 (0.12, 0.59)*	0.12 (0.05, 0.26)
SPT results		
Bermuda grass	3 (10.0%)	0
Pine	6 (20.0%)	0
Poplar	2 (4.0%)	0
Rye	2 (6.6%)	0
Timothy grass	1 (3.3%)	0
Mugwort	3 (10.0%)	0
Mite mix	30 (100%)	0
Mold mix	5 (16.7%)	0
Animal dander	3 (10.0%)	0

AR: Patients with AR. specific IgE, serum specific IgE for dust mite. The values are presented as mean ± SD or median (IQR). nd: Not detectable. *, p < 0.001 (Mann Whitney-*U* test), compared with healthy controls.
