# Supplementary material for: XBP1 is required in Th2 polarization induction in airway allergy
Source: Theranostics. 2022 Jul 11;12(12):5337–49. doi: 10.7150/thno.75100 (PMC9330522; doi:10.7150/thno.75100)
Supplement: Supplementary file 1 — Supplementary methods and figures. [file thnov12p5337s1.pdf]

Supplemental materials

Reagents

RNAi reagent kits of Esr1 and RhoA, Antibodies (Abs) of CD3 [Cat#: PC3/188A, Alexa Flour (AF) 546], CD4 (MT310, AF488), IL-13 (A130D 12G5 1E4, AF700), IL-5 (1C1, AF647), IL-4 (OX81, 8D4-8, AF594), His-tag (H-3), Flag-tag, HA-tag (F-7) were purchased from Santa Cruz Biotech (Santa Cruz, CA). MNP (3-methyl-4-nitrophenol) was purchased from Sigma Aldrich. Phospho-GATA3 (Ser308) Ab was purchased from AssayBiotech (A0933; Xiamen, China). Derf1 was purchased from Absin Bioscience (Shanghai, China). Flag-tag Ab, Derf1 Ab, ELISA kits of IL-4, IL-5, IL-13, EPX and MCP1 were purchased from DakeweBioMart (Shenzhen, China). GL7 (AF790) was purchased from BD Biosciences (Franklin Lakes, NJ). Reagents and materials for RT-qPCR, Western blotting, immunoprecipitation (IP) and chromatin IP were purchased from Invitrogen (Carlsbad, CA). Magnetic cell sorting reagent kits for immune cell isolation were purchased from Miltenyi Biotech (San Diego, CA).

Development of Th2 polarization in the mouse airways

As depicted in Fig. S2 in supplemental materials, three mouse models of airway Th2 polarization were developed. Following our established procedures (J Allergy Clin Immunol. 2019; 143:1614-1616. Eur Respir J. 2021; 57: 1902375. Immunology. 2021; 163: 310-322), three types of mouse model were developed. A, mice were treated with MNP nasal instillation daily for 2 weeks. B and C, mice were sensitized and challenged with *Dermatophagoides farinae* allergen protein-1 (Derf1) plus MNP (as an adjuvant) or ovalbumin (OVA, as an adjuvant) (detailed in Fig. S2). Th2 cytokines in nasal lavage fluids (NLF; collection procedures were referred to our previous report: J Immunol Res. 2021; 2021: 2684361) and bronchoalveolar lavage fluids [BALF; collection procedures were referred to our previous report: J Biol Chem. 2021; 296: 100585), and Th2 cell frequency in the airway tissues were assessed by FCM, and used as indicators of Th2 polarization status. The airway allergy in mice were assessed following the procedures as shown in our previous reports (J Biol Chem. 2021; 296: 100585. Immunol Lett. 2020; 228: 93-102). The experimental protocol was reviewed and approved by the Animal Ethics Committee at Longgang ENT Hospital.

Cell culture

Immune cells were cultured in RPMI1640 medium. Human kidney endothelial cells (HEK, line 293; ATCC, VA) were grown in DMEM (Dulbecco's modified Eagle's medium). Cell culture medium was supplemented with 10% fetal calf serum and 100 U/ml penicillin, 0.1 mg/ml streptomycin and 2 mM glutamine. Cell viability was greater than 99% as checked by Trypan blue exclusion assay.

Flow cytometry (FCM)

In the surface staining, cells (10^6 cells/sample) were stained with fluorochrome-labeled Abs (diluted to 1 µg/ml) or isotype IgG for 30 min at 4 °C. After washing with PBS 3 times, cells were analyzed with a flow cytometer (BD FACSCanto II). In the intracellular staining, cells were fixed with 1% paraformaldehyde (containing 0.05% Triton X-100 to increase the membrane permeability) for 1 h. After washing with PBS 3 times, cells were processed with the same procedures of the surface staining. Data were processed using a software package, Flowjo (TreeStar Inc., Ashland, OR). Data obtained from isotype IgG staining were used as gating references.

Enzyme-linked immunosorbent assay (ELISA)

Serum cytokine levels were determined by ELISA with specific reagent kits following the manufacturer's instruction.

Assessment of serum Derf1 specific IgE (sIgE) by ELISA

The levels of Derf1 specific IgE in the serum measured by using ELISA. Briefly, 96-well microtiter plates (Corning, Corning, NY, USA) were coated with Derf1 (2 µg/well), and incubated overnight at 4°C. After washing with PBST (PBS containing 0.05% Tween 20) 3 times, the plates were blocked with 5% fetal bovine serum (FBS) in PBS, and incubated for 2 h. Serum samples (diluted to 1:10; 100 µl/well) were added to each well, and was incubated for 2 hours. Biotinylated anti-mouse IgE Ab (diluted to 1:1,000), and incubated for 1 h to detect Derf1 specific IgE. After washing with PBST 3 times, the plates were incubated with streptavidin-peroxidase (diluted to 1:5,000) for 30 min at room temperature. After washing with PBST 3 times, O-phenylene diamine was added as a substrate solute; the optical densities were measured using a microplate reader (Bio-Tek Instruments, Winooski, VT, USA) at 405 nm.

Mice

C57/B6 mice (6-8-week-old) were purchased from Guangdong Experimental Animal Center. Referring to published strategies (Bettigole SE, Lis R, Adoro S, Lee AH, Spencer LA, Weller PF, et al. The transcription factor XBP1 is selectively required for eosinophil differentiation. Nat Immunol 2015; 16:829-37. Yoshinobu K, Araki M, Morita A, Araki M, Kokuba S, Nakagata N, et al. Tamoxifen feeding method is suitable for efficient conditional knockout. Exp Anim 2021; 70:91-100), *Xbp1*$^{f/f}$*Cd4*-Cre mice (here we called *Xbp1*$^{\Delta CD4}$ mice), that deleted the *Xbp1* gene in CD4$^+$ T cells, expressing Cre recombinase from the *Cd4* promoter (*Cd4*-Cre mice), were crossed with mice with loxP-flanked *Xbp1* alleles (*Xbp1*$^{f/f}$ mice). *Xbp1*$^{\Delta CD4}$ mice were bred 6 generations before using in experiments. We found that the ablation of the *Xbp1* gene in CD4$^+$ T cells did not alter the bone marrow cell numbers, successfully ablated the *Xbp1* gene in CD4$^+$ T cells, and did not alter immune cell numbers (including dendritic cells (DCs), macrophages, T cells and B cells) (Fig. S1 in supplemental

materials). Mice were maintained in a specific pathogen free facility at Shenzhen University. To activated the gene-ablation system, mice were gavage-fed with tamoxifen (200 mg/kg in corn oil) daily for 5 consecutive days before experiments.

Real-time quantitative RT-PCR (RT-qPCR)

Total RNA was extracted from cells with the TRIzol reagents, and converted to cDNA with a reverse transcription kit (Invitrogen) following the manufacturer's instruction. The cDNA samples were amplified in a qPCR device (Bio Rad CFX 96) with the SYBR Green Master Mix in the presence of relevant primers, including *XBP1* (tgtcacccctccagaacatc and aagggaggctggtaaggaac), *Xbp1* (tgtcacctccccagaacatc and aagggaggctggtaaggaac), *Il4* (aacgaggtcacaggagaagg and tctgcagctccatgagaaca). Estrogen receptor-α (*Esr1*) (gcatgatgaaaggcggcata and aaggacaaggcagggctatt). The results were calculated with the $2^{-\Delta\Delta Ct}$ method, and presented as relative expression (RE) against the house keeping gene β-actin.

Preparation of protein extracts

Cells were collected from relevant experiments and incubated with a lysing buffer (1.5 mM MgCl; 0.5 mM DTT; 10 mM HEPES; 10 mM KCl; 1 mM EDTA; 0.05% NP40 and rotease inhibitor cocktail) for 30 min. The supernatant was collected by centrifugation at 13,000 rpm for 10 min and used as the cytosolic extracts. The remained pellets were resuspended in a nuclear lysing buffer (0.2 mM EDTA; 1.5 mM $MgCl_2SO_4$; 5 mM HEPES; 4.6 M NaCl; 0.5 mM DTT; 26% glycerol) and incubated for 30 min. The supernatant was collected by centrifugation at 13,000 rpm for 10 min and used as the nuclear extracts. All the procedures were performed at 4 °C.

Western blotting

Proteins were fractioned by SDS-PAGE (sodium dodecyl sulfate polyacrylamide gel electrophoresis), transferred onto a PVDF (polyvinylidene fluoride) membrane. The membrane was blocked by incubating with 5% skim milk for 30 min, incubating with Abs of interest (Ab types are detailed in figures; diluted to 200 ng/ml) overnight at 4 °C, washed with TBST (Tris-buffered saline with 0.05% Tween 20) 3 times, incubated with secondary Abs (labeled with HRP, diluted to 20 ng/ml) of interest for 2 h, washed with TBST 3 times. Immunoblots on the membrane were developed with the enhanced chemiluminescence, and photographed in an imaging device (UVP, Cambridge, UK).

Immunoprecipitation (IP)

Pre-existing immune complexes in proteins were pre-cleared by incubating with protein G agarose beads for 2 h, and centrifuged at 5,000 *g* for 5 min. The beads were discarded. Supernatant was collected, and incubated with Abs of interest or isotype IgG overnight. Protein G agarose beads were added to the samples, and

incubated for 2 h with mild agitation. Samples were centrifuged at 5,000 *g* for 5 min. The beads were collected. Proteins on the beads were eluted, and analyzed by Western blotting.

Chromatin IP (ChIP)

Cells were fixed with 1% formalin for 15 min, lysed with a lysis buffer, followed by sonication to shear the DNA to small pieces. Samples were then processed in the same procedures of IP. Proteins/DNA were eluted from the beads. DNA was recovered with a DNA extraction kit and analyzed by PCR with the presence of *Il4* promoter primers (aacgaggtcacaggagaagg and caggacagagaaagcatcgc). The results were presented as fold change against input.

Isolation immune cells

Naïve $CD4^+$ T cells were isolated from naïve mouse spleen cells. Briefly, single spleen cells were prepared from naïve mice, from which $CD3^+$ $CD4^+$ $CD25^-$ $CD62L^+$ cells were isolated with naïve $CD4^+$ T cell isolation kit (Miltenyi) following the manufacturer's instruction. Purity of isolated $CD4^+$ T cells was greater than 95% as checked by FCM.

Th2 polarization

Naïve $CD4^+$ T cells were cultured for 3 days (the first round) in the presence of in the presence of anti-IFN-γ (10 μg/ml), IL-4 (10 ng/ml), anti-CD3 (2 μg/ml) and anti-CD28 (2 μg/ml). Cells were then collected by centrifugation, washed with culture medium, and cultured for the second round with the same reagent set as the first round.

RNA interference (RNAi)

RNAi kits of Esr1 and RhoA were purchased from Santa Cruz Biotech. $CD4^+$ T cells were isolated from the mouse spleen, and treated with reagents of Esr1 RNAi or RhoA RNAi following the manufacturer's instruction. The effects of RNAi were checked by Western blotting with the cells 48 h after the transfection.

Mass spectrometry (MS)

IP products were placed into a 1.5-ml tube. MS samples were prepared with IP products according to published protocol {Kang, 2013 #30}. DTT (20 mM) was added to the tube for protein reduction, and followed by adding iodoacetamide (50 mM) for alkylation. Trypsin was added to the samples to digest proteins at 37 °C overnight. The digested samples were desalted for LC–MS/MS analysis (AB SCIEX TOF/TOF™ 5800 system, USA). With the aid of Protein Pilot 4.0TM software (AB Sciex, USA) and referring to published procedures {Zhou, 2016 #31}, proteins were identified.

plasmids of *XBP1*, *GATA3* and *RHOA*

The plasmids of *XBP1*, *GATA3*, *RHOA* and *IL4* promoter luciferase reporter were synthesized by the Sangon Biotech (Shanghai, China). The plasmid structure information is presented in Fig. S5. Briefly, HEK293 cells were plated in a 96-well microplate (3×10^4 cells per well). One day later, plasmids were added to the wells (100 ng/well) using transfection reagent (GeneJuice; Merk Millipore) according to manufacturer's instruction. Cells were then used in further experiments.

Luciferase assay

HEK293T cells seeded on 96-well plates (3×10^4 cells/well) were transiently transfected with *IL4* promoter luciferase reporter plasmid (100 ng), with or without the co-transfection with plasmids of *GATA3*, *XBP1*, *RHOA* (100 ng each). Twenty-four hours later, the luciferase activity in the total cell lysate was measured.

Confocal microscopy

HEK293 cells were transfected with plasmids of *GATA3*, *XBP1*, *RHOA* as described above. Cells were fixed with 1% paraformaldehyde for 1 h, incubated with Abs of GATA3, XBP1 and RhoA or isotype IgG for 2 h, washed with PBS 3 times, incubated with secondary Abs [labeled with fluorochromes: Cy5 (Flag-GATA3), PE (His-XBP1), FITC (HA-RhoA)] for 2 h, washed with PBS 3 times. Cells were smeared onto a slide, mounted with cover slips, and observed with a confocal microscope (Carl Zeiss LSM710). Images were photographed at ×630.

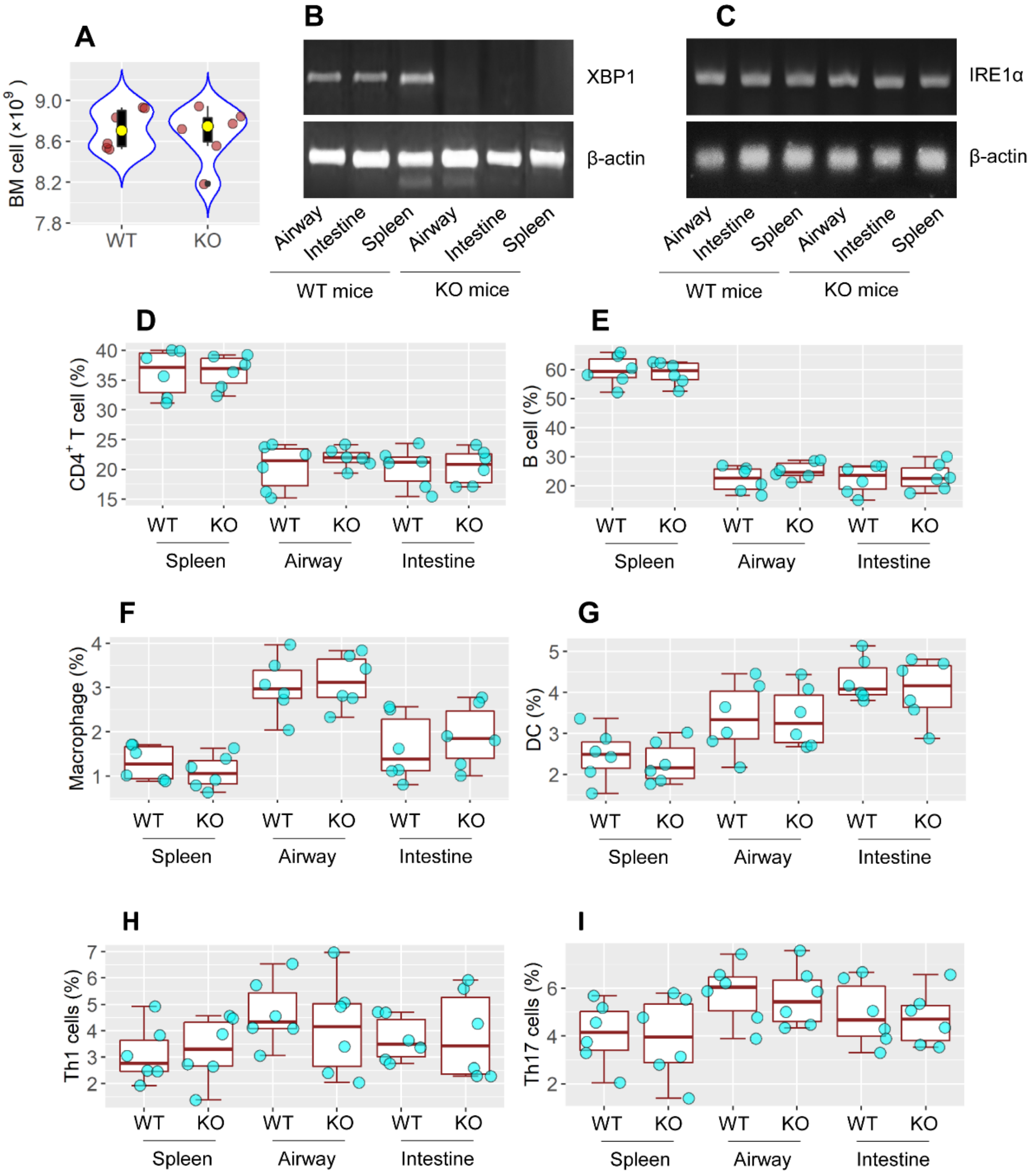

Figure S1. Assessment of immune cell frequency in WT and *Xbp1*$^{\Delta CD4}$ (KO) mice. A, violin plots show bone marrow cells of WT mice and KO mice. B-I, single cells were prepared from the airway tissues, intestinal tissues and spleen of WT mice and KO mice, and analyzed by RT-PCR and FCM. B-C, gel graphs show mRNA levels of *Xbp1* and IRE1α (representative data of 3 independent experiments). D-I, boxplots show median (IQR) of the frequency of CD4^{+} T cell (D), B cell (E), macrophage (F), DC (G), Th1 cells (H) and Th17 cells (I) from 6 mice per group. Each dot in boxplots presents data obtained from one mouse.

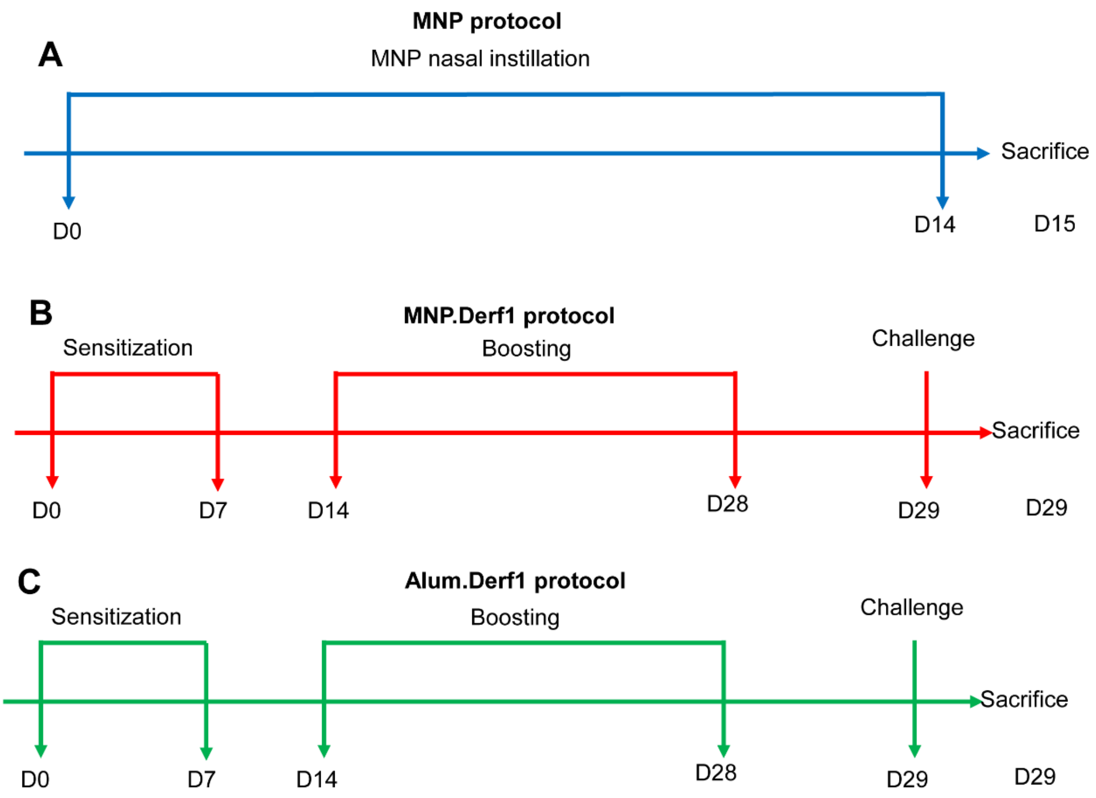

Figure S2. A scheme of the MNP protocol or MNP.Derf1 protocol.

A, MNP protocol: Purpose: To test the role of MNP in influence CD4$^+$ T cell properties. mice were treated with nasal instillation (20 µl per nostril containing MNP 1 mg/ml) daily from day 0 to day 14.

B, MNP.Derf1 protocol: Purpose: To test the effects of MNP in facilitating the development of airway allergy. Referring published procedures (Wang e et al. Allergy. 2019; 74: 1675-1690), mice were treated with the following procedures:

- **Sensitization**: Mice were injected with Derf1 (100 µg per mouse) or/and MNP (1 µM) in 0.1 ml saline through the back skin on day 0 and day 7, respectively.
- **Boosting**: Mice were treated with nasal instillation (20 µl per nostril, containing Derf1 (1 mg/ml) or/and MNP (1 µM) daily from day 14 to day 28.
- **Challenge**: Mice were treated with nasal instillation (20 µl per nostril, containing Derf1 (5 mg/ml).
  Airway allergic response was observed during 30 min after the challenge.
- **Sacrifice**: Mice were sacrificed by the cervical dislocation.

C, Alum.Derf1 protocol: Purpose: Using as a positive control. This is a conventional protocol used to develop airway allergy mouse model, including the following procedures:

- **Sensitization**: Mice were injected with Derf1 (100 µg per mouse) in 0.1 ml alum through the back skin on day 0 and day 7, respectively.
- **Boosting**: Mice were treated with nasal instillation (20 µl per nostril, containing Derf1 (1 mg/ml) daily from day 14 to day 28.

- **Challenge**: Mice were treated with nasal instillation (20 µl per nostril, containing Derf1 (5 mg/ml). Airway allergic response was observed during 30 min after the challenge.
- **Sacrifice**: Mice were sacrificed by the cervical dislocation.

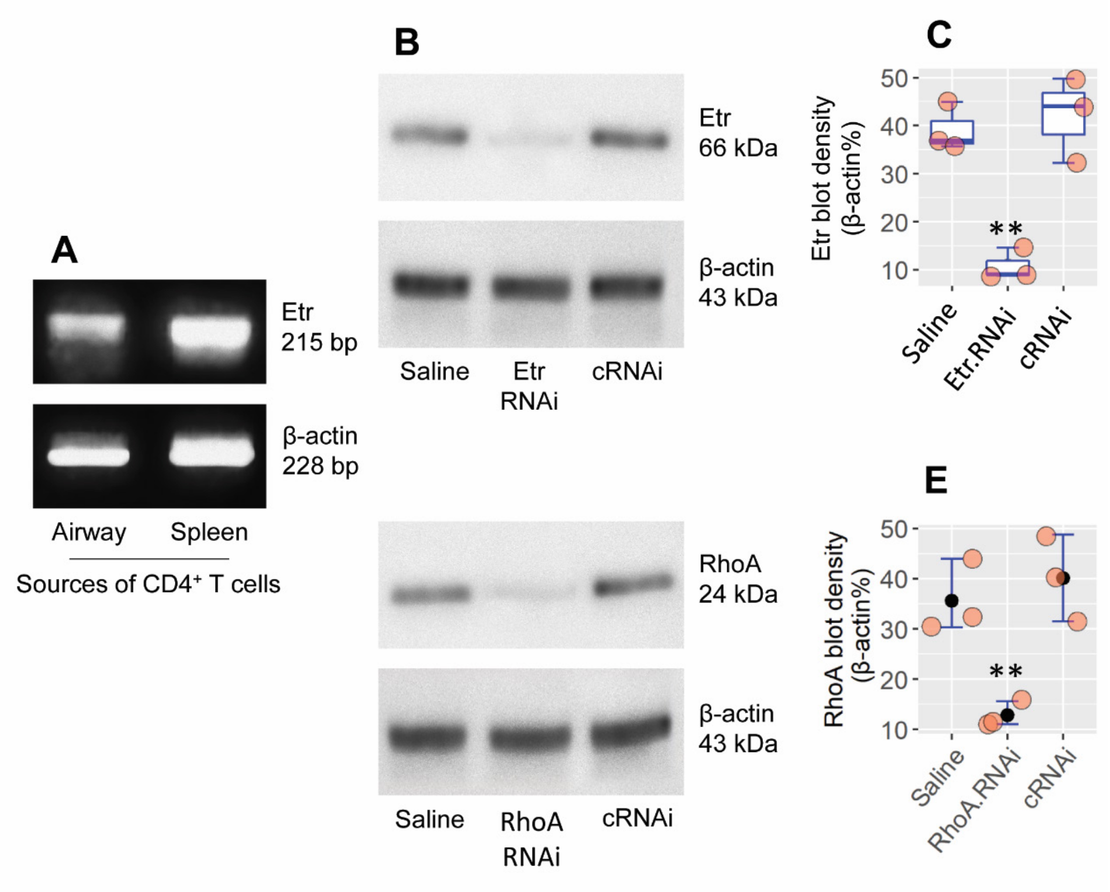

Figure S3. Depletion of estrogen receptor-α (Etr) and RhoA in CD4⁺ T cells by RNAi. A, the gel graphs show (Etr) mRNA levels in CD4⁺ T cells isolated from the naïve mouse airways and spleen. B-E, CD4⁺ T cells were isolated from the mouse spleen by MACS. CD4⁺ T cells were treated with RNAi kit of Etr or RhoA following the manufacturer's instruction. Forty-eight hours after the treatment, cells were harvested, and analyzed by Western blotting. B-C, immunoblots show Etr protein levels and boxplots show integrated density of the immunoblots in CD4⁺ T cells. D-E, immunoblots show RhoA protein levels and boxplots show integrated density of the immunoblots in CD4⁺ T cells.

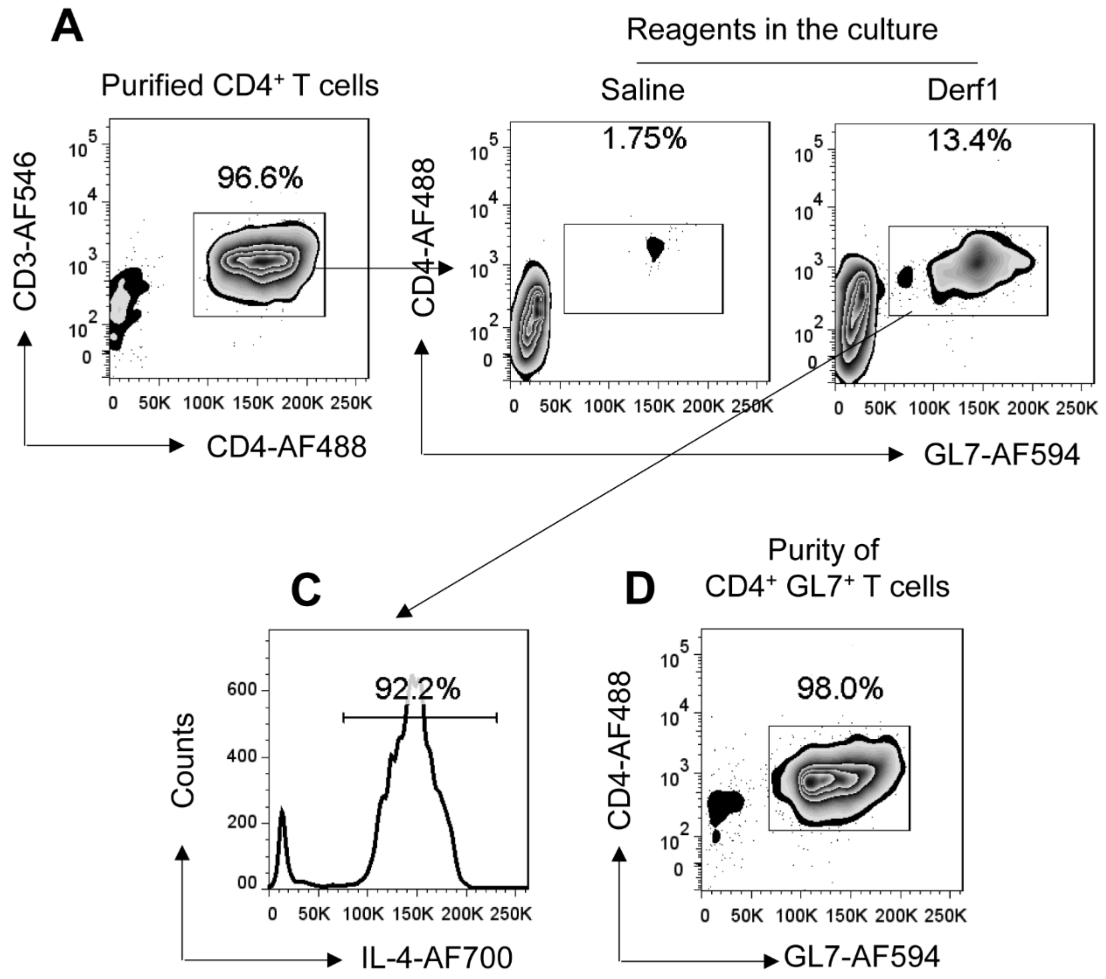

Figure S4. Purifying antigen specific Th2 cells by the specific antigen exposure protocol. Mononuclear cells were isolated from the airway tissues of mice immunized with the MNP.Derf1 protocol, from which DCs and $CD3^+$ $CD4^+$ T cells (A) were isolated by magnetic cell sorting. T cells were cultured in the presence of Derf1 (1 µg/ml) and DCs (DC:T cell = 1:10) overnight. Gated FCM plots show $GL7^+$ T cells (B; the activated T cells). The $GL7^+$ T cells are IL-4^+ (C); these cells were purified by magnetic cell sorting (D), and used as antigen-specific Th2 cells.

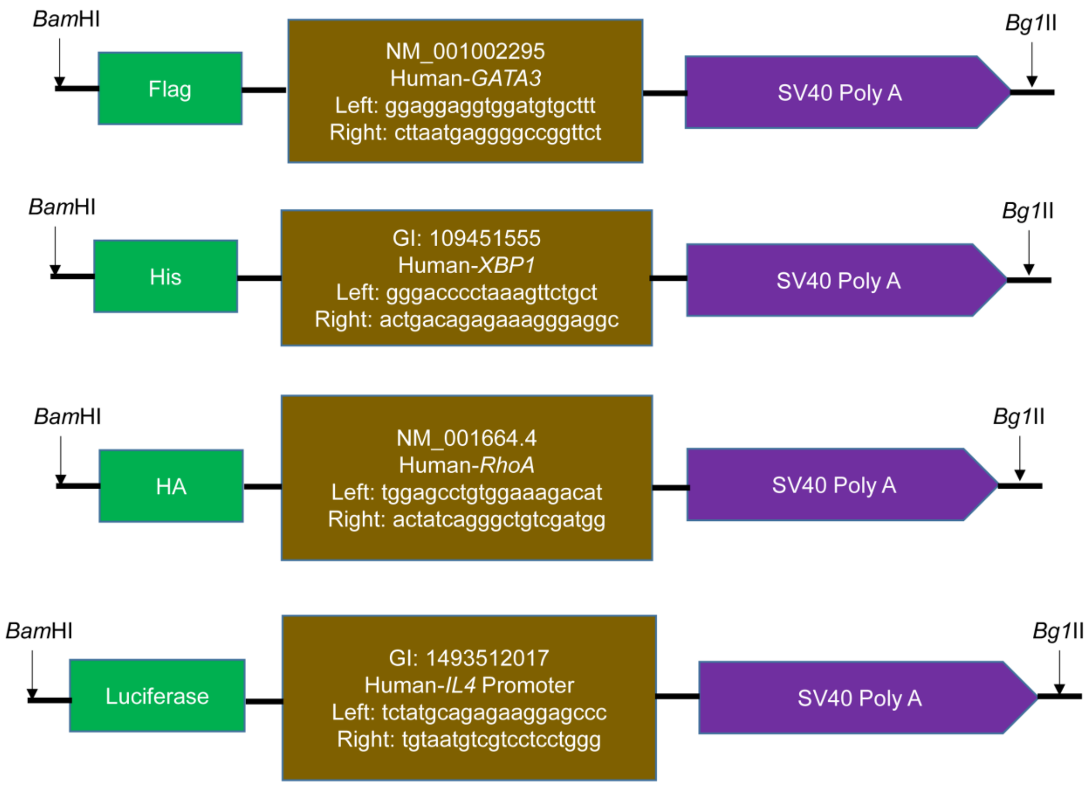

Figure S5. Schematics of plasmids used in the present study. Schematics show plasmid design (NCBI code; gene names; primers used in construction of plasmids) used in the present study, which were provided by the Sangon Biotech Company (Shanghai, China). Mutated plasmids were also constructed for each plasmid (not shown) and used in the experiments as controls (not shown).

GATA3 amino acid sequence (NCBI: NM_008091)

MEVTADQPRWVSHHHPAVLNGQHPDTHHPGLGHSYMEAQYPLTE EVDVLFNIDGQGNHVPSYY
GNSVRATVQRYPPTHHGSQVCRPPLLHGSLPWLDGGKAL SSHHTASPWNLSPFSKTSIHHGSPG
PLSVYPPASSSSLAAGHSSPHLFTFPPTPPKDVSPDPSLSTPGSAGSARQDEKECLKYQVQLPDSM
KLETSHSRGSMTTLGGASSSAHHPI TTYPPYVPEYSSGLFPPSSLLGGSPTGFGCKSRPKARSSTE
GRECVNCGATSTPLWRR DGTGHYLCNACGLYHKMNGQNRPLIKPKRRLSAARRAGTSCANCQTT
TTTLWRRNANG DPVCNACGLYYKLHNINRPLTMKKEGIQTRNRKMSSKSKKCKKVHDALEDFPKSS
SFN PAALSRHMSSLSHISPFSHSSHMLTTPTPMHPPSGLSFGPHHPSSMVTAMG

XBP1 amino acid sequence (NCBI: AF027963)

MVVVAAAPSAATAAPKVLLLSGQPASGGRALPLMVPGPRAAGSE ASGTPQARKRQRLTHLSPEEK
ALRRKLKNRVAAQTARDRKKARMSELEQQVVDLEEEN HKLQLENQLLREKTHGLVVENQELRTRL
GMDTLDPDEVPEVEAKGSGVRLVAGSAESA ALRLCAPLQQVQAQLSPPQNIFPWTLTLLPLQILSLI
SFWAFWTSWTLSCFSNVLPQS LLVWRNSQRSTQKDLVPYQPPFLCQWGPHQPSWKPLMNSFV
LTMYTPSL

RhoA amino acid sequence (NCBI: AF498970.1)

MAAIRKKLVIVGDGACGKTCLLIVFSKDQFPEVYVPTVFENYVADIEVDGKQ
VELALWDTAGQEDYDRLRPLSYPDTDVILMCFSIDSPDSLENIPEKWTPE
VKHFCPNVPIILVGNKKDLRNDEHTRRELAKMKQEPVKPEEGRDMANRIG
AFGYMECSAKTKDGVREVFEMATRAALQARRGKKKSGCLVL

Figure S6. Identification of GATA3, XBP1 and RhoA in IP products by MS. Antigen-specific Th2 cells were isolated from airway tissue mononuclear cells, which were processed by IP with an anti-GATA3 Ab as a bait. The IP products were analyzed by MS. Representative peptides (highlighted in red) are presented in the amino acid sequences of GATA3, XBP1 and RhoA, respectively.

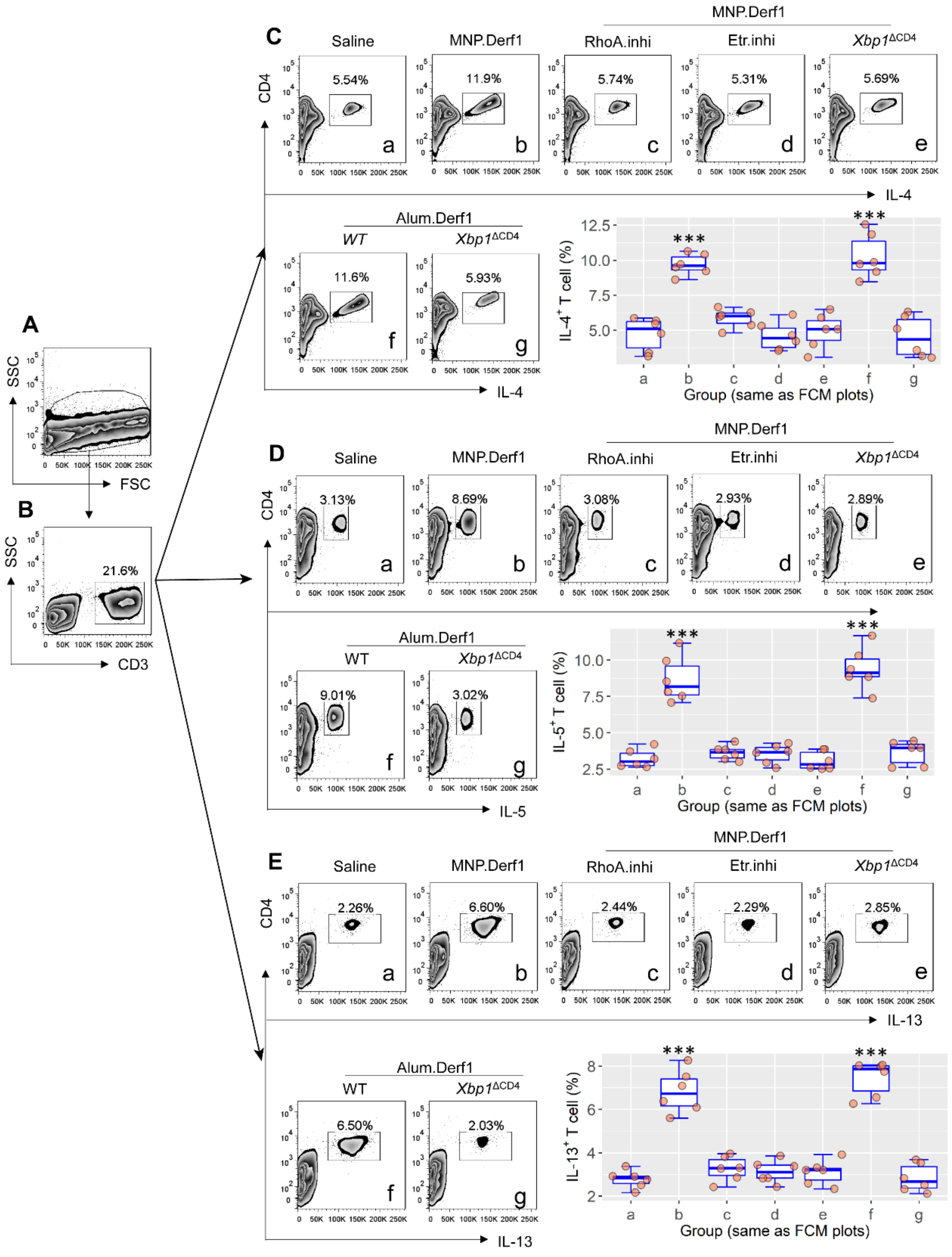

Figure S7. Assessment of Th2 cell frequency in the airway tissues. WT mice and *Xbp1*$^{\Delta CD4}$ mice were sensitized with the MNP.Derf1 protocol or the Alum.Derf1

protocol (Fig. S2) with the treatment denoted above FCM plots. AMCs were prepared with the airway tissues, and analyzed by FCM. A, the FSC/SSC plots. B, $CD3^+$ T cells were gated. C-E, from $CD3^+$ T cells, $CD4^+$ $IL\text{-}4^+$ T cells (C), $CD4^+$ $IL\text{-}5^+$ T cells (D), $CD4^+$ $IL\text{-}13^+$ T cells (E) were gated. Boxplots show median (IQR) of Th2 cell counts from 6 mice per group. *** ($p<0.001$), compared with group a. WT: Wild type. *Xbp1*$^{\Delta CD4}$ mice: Mice carry *Xbp1*-deficient $CD4^+$ T cells. MNP: 3-methyl-4-nitrophenol. Derf1: D. *farinaeallergen* protein 1. AMC: Airway mononuclear cell. FCM: Flow cytometry. Etr.inhi ("inhi" stands for "inhibitor"): Etr inhibitor. Mice were treated with nasal instillation containing Bay-2416964 (1 µM). RhoA.inhi: Mice were treated with nasal instillation (20 µl/nostril, containing RhoA inhibitor, Bay-293 40 nM) daily during the sensitization period.
